# OTEC Maximum Net Power Output Using Carnot Cycle and Application to Simplify Heat Exchanger Selection

**DOI:** 10.3390/e21121143

**Published:** 2019-11-22

**Authors:** Kevin Fontaine, Takeshi Yasunaga, Yasuyuki Ikegami

**Affiliations:** 1 Graduate School of Science and Engineering, Saga University, 1 Honjo-Machi, Saga 840-8502, Japan; 2 Institute of Ocean Energy, Saga University, 1 Honjo-Machi, Saga 840-8502, Japan; yasunaga@ioes.saga-u.ac.jp (T.Y.); ikegami@cc.saga-u.ac.jp (Y.I.)

**Keywords:** ocean thermal energy conversion (OTEC), plate heat exchanger, optimization, maximum power output, finite-time thermodynamics

## Abstract

Ocean thermal energy conversion (OTEC) uses the natural thermal gradient in the sea. It has been investigated to make it competitive with conventional power plants, as it has huge potential and can produce energy steadily throughout the year. This has been done mostly by focusing on improving cycle performances or central elements of OTEC, such as heat exchangers. It is difficult to choose a suitable heat exchanger for OTEC with the separate evaluations of the heat transfer coefficient and pressure drop that are usually found in the literature. Accordingly, this paper presents a method to evaluate heat exchangers for OTEC. On the basis of finite-time thermodynamics, the maximum net power output for different heat exchangers using both heat transfer performance and pressure drop was assessed and compared. This method was successfully applied to three heat exchangers. The most suitable heat exchanger was found to lead to a maximum net power output 158% higher than the output of the least suitable heat exchanger. For a difference of 3.7% in the net power output, a difference of 22% in the Reynolds numbers was found. Therefore, those numbers also play a significant role in the choice of heat exchangers as they affect the pumping power required for seawater flowing. A sensitivity analysis showed that seawater temperature does not affect the choice of heat exchangers, even though the net power output was found to decrease by up to 10% with every temperature difference drop of 1 °C.

## 1. Introduction

In 2015, the CO_2_ emission due to energy generation and heat production was up to 13 540 million tons [1]. Efforts are to be made to lower this figure, especially to meet the Paris Agreement’s goal to limit global warming to below 2 °C [2]. Thus, it is necessary to develop renewable energies, which are not represented enough in today’s energy mix [3]. A drawback of most implemented renewable energies is their intermittency, therefore, they cannot be used for baseload energy demand without a storage system breakthrough. However, to generate electricity, ocean thermal energy conversion (OTEC) uses the difference between the surface seawater and the deep seawater temperature in tropical areas. As such areas present very low temperature change throughout the year, a steady power generation can be achieved. Moreover, OTEC has huge potential, as its resources are estimated at a maximum of 7 TW of net energy production [4]. In addition to power generation, it is possible with such a system to produce freshwater using the warm seawater. This water can be used to create pure hydrogen to store or transport the energy generated by the power plant. In addition to tackling climate change and providing clean energy, OTEC can contribute to reaching five other sustainable development goals defined in 2015 by the general assembly of the United Nations [5]. Indeed, OTEC produces deep seawater as a byproduct, which can be used for aquaculture and desalination. The implementation of such a power plant could play a major role in the economic growth of cities and countries, especially on islands. It would also promote industry, innovation, infrastructure, as well as providing employment for the construction and operation of the plant, auxiliaries, and other industries that make use of deep seawater.

As it uses the low-temperature gradient in the ocean, which is 20–25 °C in suitable locations, the OTEC system presents a very low maximal theoretical thermal efficiency of 3%–5%. However, the system is a potential substitute for conventional power plants with higher efficiency, considering that the resource has virtually no cost. It is necessary to understand the basic theory, known as finite-time thermodynamics (FTT), which was first applied to OTEC by Wu [6] and extended by Ikegami and Bejan [7]. They constructed the basic theory for OTEC using FTT; however, the application in engineering is still limited. It is also necessary to optimize, as much as possible, the net power output of an OTEC power plant. This can be done in different ways: by optimizing cycles or investigating more efficient ones, by choosing the most suitable working fluid, or by investigating key elements of the system.

To harvest ocean thermal energy, it is possible to use a flash chamber to evaporate the warm surface seawater. The steam is used to operate a turbine and is then condensed by the cold deep seawater. In doing so, both energy and freshwater are produced. Kim et al. researched such a system, namely, open-cycle OTEC [8]. They thoroughly investigated the system using three different condensers in order to adjust the power generation and desalination. They found that there is an optimal fraction of steam that enters the turbine that maximizes the ratio of the generated power over the cold water flow rate, therefore optimizing the use of the said steam throughout the plant. Kim et al. also investigated the possible replacement of the flash evaporation chamber by a vacuum membrane distillation module, which would require less than 10% of the volume normally occupied by the evaporator and result in a decrease of the open-cycle OTEC plant size by 30% and the electricity cost by 2.1% [9]. In contrast to the open cycle, the closed cycle uses a working fluid in a closed-loop, and is the main focus of this study, as it is more suitable to generate large amount of power. Hereafter, only the closed cycle is discussed.

Regarding cycle investigations, D.H. Johnson calculated the exergy available in a specific amount of both cold and warm water and used the results to compare the performance of different OTEC cycles, such as the Beck and Rankine cycles. He used the second law efficiency of the cycles, defined as the ratio between the cycle power output and the exergy from ocean thermal resources [10]. His study concluded the triple Rankine cycle as the one which presents the most potential. He also compared the second law efficiency of a coal-fired Rankine cycle with a single-stage OTEC Rankine cycle and found that “an OTEC plant uses the exergy of the ocean thermal resource about as efficiently as a conventional coal-fired plant uses the exergy of coal”. In 1982, Kalina presented a cycle that introduced the regeneration of the working fluid [11]. This cycle has been widely investigated: Zhang et al. made a review of this research and realized a comparison with the Rankine cycle [12]. They found that, in most cases, the Kalina cycle presents better efficiencies than the Rankine cycle, especially in the case of low-temperature resources. Uehara et al. then proposed a new cycle, as an improvement of the Kalina cycle, and implemented a second turbine to the cycle [13]. The study showed an increase of ~10% in the thermal efficiency compared to a Kalina cycle and more than 30% compared to a single-stage Rankine cycle for specific conditions. Ikegami et al. investigated the reduction of irreversible losses in the heat exchange process for a double-stage Rankine cycle [14]. This way, they demonstrated that an OTEC cycle net power output could be increased using this cycle or the Kalina cycle. The current study initially focused on the Carnot cycle, as it is the basic theoretical cycle for OTEC. After that, a Rankine cycle was investigated, as it is a more realistic cycle and for comparison purposes. Additionally, calculations from the Rankine cycle can be used as a base for multistage Rankine cycle calculations in the future, as they have been found to have great potential.

For fluid choices, Dijoux et al. built a model of an organic Rankine cycle applied to OTEC to compare the performances of 26 different working fluids. They managed to select three fluids that would be suitable for OTEC by considering several parameters, including thermodynamics performances, even though no fluid could meet all the ideal parameters [15]. One of the selected fluids was ammonia, which has also been identified as the fluid of choice by Bernardoni et al. [16]. Thus, this study considered ammonia when working fluid properties were required.

In OTEC, heat exchangers allow heat harvest and are pointed out by many studies as one of the most important parts of the system to investigate. Sinama et al. performed an OTEC cycle optimization by minimizing the destroyed exergy [17]. The study presents a sensitivity analysis that shows the role of heat exchangers in an OTEC cycle as well as the importance of optimizing them or developing more suitable ones. Sun et al. reached the same results in their optimization design and exergy analysis of an organic Rankine cycle [18]. Bernardoni et al. realized a techno-economic analysis of a closed OTEC cycle to assess its levelized cost of energy (LCOE) [16]. This is defined by the sum of the operating cost of a power plant over its lifetime and its capital cost divided by the net energy production over its lifetime. Bernardoni et al. identified heat exchangers as one of the most expensive constituents of an OTEC power plant, making them suitable candidates for further study. Accordingly, this paper focused on investigating heat exchangers for OTEC.

Many types of heat exchangers exist, and the most investigated one in OTEC is the plate heat exchanger. Indeed, the compactness of this type of heat exchanger compared to other types allows reaching the significant heat transfer area required for OTEC purposes with less material. It is necessary to compare the maximum net power output of a power plant for different heat exchangers to choose the most suitable one. For this purpose, it is first necessary to evaluate the net power output of an OTEC system for a given heat exchanger, which depends not only on the heat transfer performance of the heat exchanger but also its pressure drop. Due to the low thermal efficiency of the OTEC cycle, the pumping power required to counter the pressure drop that occurs inside the heat exchanger is of the same order of magnitude as the gross power generated by the harvested heat. Yasunaga et al. [19] realized an OTEC performance evaluation in terms of FTT based on a Carnot cycle and identified a theoretical relationship between heat transfer performance, pressure drop, and OTEC net power output. They also showed that a compromise must be found, as both parameters are not independent. To evaluate the net power output of an OTEC power plant, it is thus necessary to know both the heat transfer performance and the pressure drop of the heat exchanger. Although they are independently evaluated, both these parameters have been widely investigated [20,21,22]. Uehara and Ikegami conducted an in-depth optimization of a closed-cycle OTEC system with all its constituents for different temperatures of the warm seawater [23]. They used the ratio of the heat transfer area over the cycle net power output as the objective function to minimize the cost of the produced electricity, which greatly depends on the size of the heat exchangers. In doing so, they could calculate, among other things, the optimized, required heat transfer area for a specific evaporator and condenser set. However, studies that have evaluated an OTEC system net power output as a function of both the heat transfer performance and the pressure drop, to allow a comparison between different heat exchangers, are scarce. The method used by Uehara et al., although very accurate and quite exhaustive, is not suitable for heat exchanger comparisons due to its high complexity. It is therefore necessary to develop a relatively easier method that will allow such a comparison. This was the aim of the present study, and the main focus was on the trade-off between the pressure drop and the heat transfer performance; losses occurring on other components should not affect the comparison results.

The goal of this work was to find the most suitable heat exchanger for OTEC, which is useful when designing a new power plant. A simplified method was developed to evaluate and then maximize the net power output of an OTEC system for a given heat exchanger. As it is one of the most expensive components, it is necessary to select the heat exchanger that leads to the highest net power output for a minimal amount of material [16,17,18]. This study, therefore, focused on the net power output per unit of the heat exchanger surface area. Increasing the heat transfer area on plate heat exchangers, the preferred type of heat exchanger for OTEC, can be easily achieved by adding more plates. This will not alter the results as long as the Reynolds numbers inside a plate and the number of passes remains the same. The results for three different heat exchangers from the literature were compared. First, calculations were done for the Carnot cycle. Then, Rankine cycle calculations were realized to investigate the differences between the two cycles. The influence of seawater temperature was investigated through a sensitivity analysis. Indeed, each OTEC power plant is designed to operate at specific conditions, and the heat source temperature affects their performance. This method will not be able to predict the net power output and optimum operating points as accurately as what was achieved by Uehara and Ikegami [23]; however, its relative ease of use will make it preferable for heat exchanger comparisons. In addition, it may constitute the basis for less complex methods than the one proposed by Uehara and Ikegami, but with comparable accuracy. The proposed method presents the following:

- It addresses the lack of evaluation methods to efficiently select a heat exchanger for OTEC purposes and considers the trade-off between heat transfer performance and pressure drop.
- It is easily applicable for different heat exchangers as long as geometry, heat transfer coefficient correlation, and pressure drop correlation are provided.
- It is easily applicable to different seawater temperatures.

## 2. Description and Analysis

The first step before the actual optimization is to define the objective function that will be maximized. In this study, the comparison of the heat exchangers was based on the net power output of the power plant per unit of the heat exchanger surface area. The net power output of an OTEC system depends on the gross power output that can be produced by the harvested heat and the different losses that occur in the system.

In OTEC, the heat from the sea is harvested using plate heat exchangers. A plate heat exchanger consists of a stack of plates in which the thermal energy of a relatively hot fluid is transferred to a colder one, with each fluid flowing on a different side of a plate. Therefore, the harvested heat depends on the convection heat transfer coefficient of both fluids and the heat transfer conductivity of the plate through which the heat transfer occurs. In addition, deposit layers of different natures are observed on the heat exchanger plate as the OTEC plant operates. This is called fouling and is caused by elements; organic matter; or living organisms present in the seawater. Such layers add thermal resistance to the plate, and thus decrease the heat transfer performance of the exchanger.

Part of the gross power output generated by the power plant is used for water ducting. In addition, losses happen in the heat transfer process itself and in the form of pressure drop that will be countered using pumps, decreasing the net power output of the system. The pressure drop happens in the pipes in which each fluid circulates and in the heat exchangers.

In this study, an optimization was realized for an OTEC system based on a Carnot and a Rankine cycles. For both cycles, the following assumptions were considered:

- The heat transfer coefficient of the working fluid is much greater than the seawater one as the working fluid undergoes a phase change [24].
- The thermal resistance due to fouling can be neglected.
- The pressure drop on the working fluid side can be neglected [16,17,23].
- Changes in the water thermodynamic properties in the heat exchangers due to temperature variation can be neglected.

Moreover, although it has a non-negligible impact on the net power output of an OTEC power plant, the pressure drop that occurs in the piping system is not accounted for in the present paper. Indeed, it mainly depends on the length of the pipes and therefore, it has no impact on the choice of a heat exchanger.

### 2.1. Carnot Cycle: Concept and Equations

**Figure 1:**
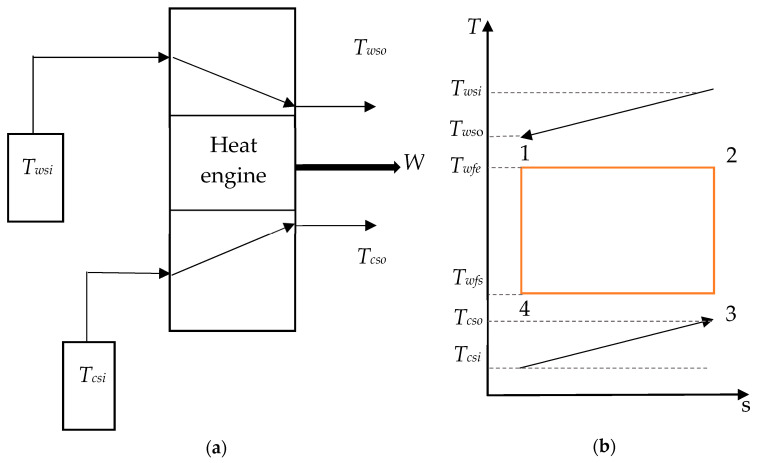
(**a**) Model of the ocean thermal energy conversion (OTEC) system and (**b**) temperature–entropy diagram of a Carnot cycle.

In this section, the conceptual OTEC system in Figure 1a is assumed to be working on a Carnot cycle, which is described in Figure 1b. A working fluid is heated by the warm seawater in a first heat exchanger in which it evaporates. The resulting vapor is then used to operate a turbine before being condensed by the deep seawater through a second heat exchanger. Finally, the working fluid is pumped into the evaporator for another cycle. This is an ideal cycle using isothermal heat exchange as well as isentropic compression and expansion processes, although they cannot be achieved in a practical power plant.

As this study focuses on the cycle performance, the pumping power required for water ducting is not considered. Thus, the net power output is equal to the difference between the gross power generated by the heat engine, *W_gross_*, and the required pumping power to counter the seawater pressure drop in the heat exchanger, *W_P_*. The net power output, *W_net_*, of the Carnot cycle is therefore given by Equation (1) [7]:

(1) $$W_{net}=W_{gross}-W_{P}$$

The power generated by the cycle is given by the balance between the heat added to the system (*Q_e_*) and the heat taken from the system (*Q_c_*) Equation (2) [25]:

(2) $$\begin{array}{cc} W_{gross}=Q_{e}-Q_{c} & =C_{ws}\epsilon_{ws}\left(T_{wsi}-T_{wfe}\right)-C_{cs}\epsilon_{cs}\left(T_{wfc}-T_{csi}\right)\\ & ={\left(mc_{p}\epsilon \right)}_{ws}\left(T_{wsi}-T_{wfe}\right)-{\left(mc_{p}\epsilon \right)}_{cs}\left(T_{wfc}-T_{csi}\right) \end{array}$$

where *ε* is the heat exchanger efficiency, which is equal to the ratio of the actual heat exchange that occurs in the heat exchanger and the maximum theoretical heat exchange that could occur for an infinite counterflow heat exchanger without losses. *C* is the product of the mass flow rate, *m*, and the specific heat capacity, *c_p_*, of the seawater. Subscript *ws* stands for warm source and *cs* stands for cold source. *T_wsi_*, *T_csi_*, *T_wfe_*, and *T_wfc_* are the temperature of the warm seawater at the inlet of the evaporator, the temperature of the cold seawater at the inlet of the condenser, the temperature of the working fluid in the evaporator, and the temperature of the working fluid in the condenser, respectively.

In the case of an OTEC power plant, as a phase change occurs in both heat exchangers, the efficiency, *ε*, can be written as Equations (3) and (4) [25]:

(3) $$\epsilon_{\mathrm{ws}}=1-\exp\left(-{\mathrm{NTU}}_{\mathrm{ws}}\right)$$

(4) $$\epsilon_{\mathrm{cs}}=1-\exp\left(-{\mathrm{NTU}}_{\mathrm{cs}}\right)$$

where NTU is the number of transfer units and is defined in Equation (5):

(5) $$\mathrm{NTU}=\frac{UA}{C}$$

where *U* is the overall heat transfer coefficient and *A* is the heat transfer area of the heat exchanger.

The Lagrange multiplier method is applied, similar to Ibrahim et al., to find the conditions on the temperatures that maximize the power output of the heat engine, as can be seen from Equations (6) to (9) [26]. The entropy balance written in Equation (6) is used as the constraint function, as it is equal to 0 when there are no irreversibilities:

(6) $$\Delta s=\frac{C_{ws}\epsilon_{ws}\left(T_{wsi}-T_{wfe}\right)}{T_{wfe}}-\frac{C_{cs}\epsilon_{cs}\left(T_{wfc}-T_{csi}\right)}{T_{wfc}}=0$$

The Lagrange multiplier, *Λ*, is then introduced and verifies Equation (7):

(7) $$\frac{\delta W_{gross}}{\delta T_{wfe}}=\Lambda \frac{\delta \Delta s}{\delta T_{wfe}}\;\mathrm{and}\;\frac{\delta W_{gross}}{\delta T_{wfc}}=\Lambda \frac{\delta \Delta s}{\delta T_{wfc}}$$

It leads, using Equations (2), (6), and (7), to the following relationship at the maximum point.

(8) $$\frac{T_{wfc}}{T_{wfe}}=\sqrt{\frac{T_{csi}}{T_{wsi}}}$$

From here, it is possible to express the maximum power output of the heat engine, as in [7,19,26]:

(9) $$W_{gross}=\frac{{\left(\sqrt{T_{wsi}}-\sqrt{T_{csi}}\right)}^{2}}{\frac{1}{C_{ws}\epsilon_{ws}}+\frac{1}{C_{cs}\epsilon_{cs}}}$$

In the case of OTEC, as heat exchangers are the most expensive components [16], the goal of optimization is to maximize the net power output per unit of heat exchanger surface area. Therefore, the objective function is defined as the net power output of the heat engine, assuming it operates at the optimized temperature ratio shown in Equation (8) [7], divided by the total surface area of both the evaporator and condenser. In addition, heat exchangers, for which the calculation is made, do not have the same surface area. Thus, given that the surface area can be changed by the addition or subtraction of plates, the net power output per unit of surface area, $w_{net}$, allows a more efficient comparison. It is expressed in Equation (10), which is deduced from Equations (1) and (9):

(10) $$w_{net}=\frac{1}{\left(As_{ws}+As_{cs}\right)}\left(\frac{{\left(\sqrt{T_{wsi}}-\sqrt{T_{csi}}\right)}^{2}}{\frac{1}{C_{ws}\left(1-e^{-{\mathrm{NTU}}_{\mathrm{WS}}}\right)}+\frac{1}{C_{cs}\left(1-e^{-{\mathrm{NTU}}_{\mathrm{WS}}}\right)}}-W_{P}{}_{ws}-W_{P}{}_{cs}\right)$$

where *As* is the heat exchanger surface area calculated as As=LWi(number of plates−2), with *L* and *Wi* representing the length and width of the plate, respectively. *As* differs from *A* as it does not take into account any plate surface patterns, such as herringbone. *W_P_* is defined as in [7]:

(11) WP=2fLRe3μw3SD4ρw2

where *D* is the equivalent diameter of the heat exchanger, *L* is the length of a plate and the Reynolds number, Re, is defined as:

(12) Re=ρwvDμw

with *ρ_w_* the seawater density, *v* the seawater mean velocity, and *µ_w_* the seawater dynamic viscosity.

*f* is the friction factor and is defined as:

(13) $$f=\frac{\tau }{\frac{\rho_{w}v^{2}}{2}}$$

where *τ* is the wall shear stress of the heat exchanger. However, in this study, an experimental correlation of the friction factor is used. It is assumed that the friction factor can be written as:

(14) f=βReξ

where *β* and *ξ* are two constants that depend on the heat exchanger properties.

*UA*, from Equation (5), is defined as follows:

(15) $$\frac{1}{UA}=\frac{1}{\alpha_{w}A}+\frac{t}{\lambda_{p}A}+\frac{1}{\alpha_{wf}A}+\frac{R_{f}}{A}$$

where *α_w_* is the seawater convective heat transfer coefficient, *α_wf_* is the working fluid heat transfer coefficient, *t* is the plate thickness, *R_f_* is the resistance due to fouling, and *λ_p_* is the thermal conductivity of the plate.

As specified at the beginning of Section 2, fouling is neglected and *α_wf_* is assumed to be much greater than *α_w_*. Therefore:

(16) $$\frac{1}{\alpha_{w}A}+\frac{t}{\lambda_{p}A}+\frac{1}{\alpha_{wf}A}+\frac{R_{f}}{A}\approx \frac{1}{\alpha_{w}A}+\frac{t}{\lambda_{p}A}=\frac{1}{\alpha_{w}A}+B$$

where *B* is a constant and *α_w_* is calculated from the Nusselt number (Nu), defined as follows, with *λ_w_* being the water thermal conductivity and *D* the equivalent diameter:

(17) $$\mathrm{Nu}=\frac{\alpha_{w}D}{\lambda_{w}}.$$

Furthermore, the following assumption is taken:

(18) $$\mathrm{Nu}=dRe^{\gamma }Pr^{n},$$

with *d*, *γ*, and *n* being constant coefficients for the Nusselt correlation. The Prandtl number, Pr, is defined as:

(19) $$\mathrm{Pr}=\frac{\mu_{w}c_{p}}{\lambda_{w}},$$

leading to:

(20) UA=ANuλwD+BANuλw=AdReγPrnλwD+BAdReγPrnλw

In addition, using Prandtl and Reynolds numbers definitions from Equations (12) and (19), *C* can be written as:

(21) $$C=mC_{p}=\rho vSC_{p}=\frac{\mathrm{RePr}\lambda_{w}S}{D}$$

Therefore,

(22) NTU=UAC=DRePrλwSAdReγPrnλwD+BAdReγPrnλw=dReγ−1Prn−1AD(D+BdReγPrnλwA)S

Then, replacing NTU and *C* in Equation (10) by their respective expressions described in Equations (22) and (21), the following equation of the net power output per unit of heat exchanger surface area, *w_net_*, is deduced:

(23) wnet=1Asws+Ascs(Tws−TCS)21(RePrλSD(1−exp(−dReγ−1Prn−1AD(D+BdReγPrnλA)S)))WS+1(RePrλSD(1−exp(−dReγ−1Prn−1AD(D+BdReγPrnλA)S)))CS−1Asws+Ascs((2fLRe3μ3SD4ρ2)WS−(2fLRe3μ3SD4ρ2)CS)

It has been possible here to express the net power output of the heat engine as a function of the properties of the seawater side only, which will facilitate the calculations.

As the Carnot cycle is ideal, it does not reflect the actual net power output of an OTEC system. It is possible to introduce a FTT irreversibility factor, as done by Ibrahim et al. [26]; however, this coefficient regroups different sources of internal irreversibilities, which makes it difficult to assess, and is therefore not usable for practical applications like the OTEC power plant. A better way is to proceed with a cycle that can be used for OTEC, such as the Rankine cycle. A comparison must be carried out to see if the use of a different cycle will have an impact on the choice of a heat exchanger.

### 2.2. Rankine Cycle: Concept and Equations

**Figure 2:**
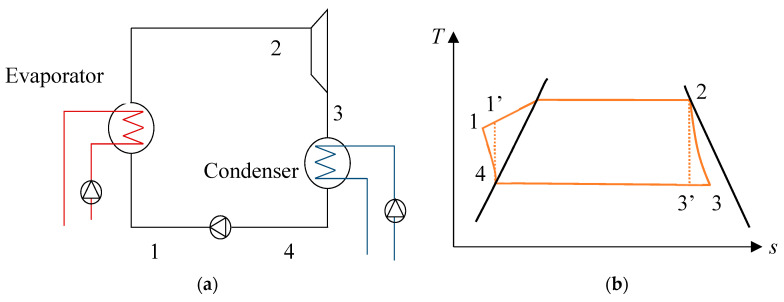
(**a**) Description of the Rankine cycle and (**b**) temperature–entropy diagram of a Carnot cycle.

For the Rankine cycle, described in Figure 2a,b, the net power output can be given by Equation (24). It was assumed that the working fluid at the outlet of the heat exchangers was in a saturated state. This cycle differs from the Carnot cycle, as it can be used practically in power plants. In this case, heat exchange is not isothermal, and a change in entropy occurs in both compression and expansion processes.

As for the Carnot cycle, the objective function is the net power output of the heat engine divided by the total heat exchanger surface area:

(24) wnet=1Asws+Ascs(mwf(h2−h3−h1+h4)−(2βRe3+ξLμ3SD4ρ2)ws−(2βRe3+ξLμ3SD4ρ2)cs)

where *m_wf_* is the mass flow rate of the working fluid. *h*_1_, *h*_2_, *h*_3_, and *h*_4_ are the enthalpy values of the corresponding points in Figure 2 and with:

(25) $$h_{3}=h_{2}\left(1-\eta_{T}\right)+\eta_{T}h_{3}^{\prime }\;and\;h_{1}=h_{4}\left(1-\eta_{p}\right)+\eta_{p}h_{1}^{\prime }$$

*h’*_1_ and *h’*_3_ are the enthalpy values of the corresponding points in the case of an isentropic process, and *η_T_* and *η_p_* are the turbine and pump efficiencies, respectively. For the calculations, the turbine efficiency is equal to 0.85 and the pump efficiency is equal to 0.8.

Enthalpy values can be calculated from temperatures *T*_2_ and *T*_4_ at Points 2 and 4 using the software REFPROP [27]. *T*_2_ and *T*_4_ can be evaluated from Equation (26):

(26) $$T_{2}=\frac{T_{wsi}-T_{wso}e^{{\mathrm{NTU}}_{\mathrm{ws}}}}{\epsilon_{ws}}\;and\;T_{4}=\frac{T_{csi}-T_{cso}e^{{\mathrm{NTU}}_{\mathrm{cs}}}}{\epsilon_{cs}}$$

*h*_2_ and *h*_4_ are calculated using the respective temperature and because the fluid is at a saturated state. It is possible to calculate *h*_3_ using *T’*_3_ = *T*_4_, *s*_2_ = *s’*_3_, and Equation (26). For Point 1, *s*_4_ = *s’*_1_ and *P*_2_ = *P’*_1_; *P* is the pressure of the fluid and *s* is its specific entropy. NTU is calculated as detailed for the Carnot cycle in Equations (15)–(22). Details for the calculations to obtain Equation (26) are given in Appendix A.

## 3. Optimization Process

The optimization was realized for different plate heat exchangers tested by Kushibe et al. [28]. The specifications are given in Table 1; they are all versatile plates. Plate heat exchanger (PHE) 1 is meant for high pressure and high temperature and is used as an evaporator in power generation systems using hot springs as a heat source, PHE 2 is a typical herringbone plate heat exchanger, and PHE 3 was specially invented by Prof. Uehara as both an evaporator and a condenser [28].

The optimization was run using the “minimize” function written by Rody Oldenhuis based on the derivative-free method (Nelder–Mead simplex algorithm) fminsearch function of Matlab R2017a (MathWorks, Natick, MA, USA) [29]. It searches for a local minimum of a given objective function with several variables. It allows applying boundaries on the variables as well as linear and nonlinear equality and inequality constraints that the objective function needs to satisfy. In the present work, boundaries were specified for all the variables and calculations were performed several times from different starting points generated randomly within the boundaries so that a global minimum could be found. As the goal was to maximize *w_net_*, the objective function used with “minimize” was −*w_net_*.

Calculations were done using the friction factor and Nusselt numbers correlations of the corresponding heat exchanger, as defined in Equations (14) and (18). Table 2 gives the different coefficients for the correlations, which were approximated from linear interpolation from the data of the study realized by Kushibe et al. [28].

For both cycles, the water properties are taken at pressures of 130 kPa and 170 kPa for warm and cold seawater sources, respectively, for the corresponding temperatures and were obtained using REFPROP [27]. Warm and cold water source temperatures are first set at 30 °C and 5°, respectively. Then, a sensitivity analysis is performed for both cycles with a temperature difference between 23 °C and 20 °C. These temperature differences are obtained by decreasing the warm and cold water source alternatively.

### 3.1. Carnot Cycle

In the case of the Carnot cycle, the objective function from Equation (23) presents only two variables: the two Reynolds numbers. They were limited to a corresponding water mean velocity between 0.2 m/s and 1.8 m/s, which is the speed range reachable in plate heat exchangers. No constraints were applied to the Carnot cycle.

### 3.2. Rankine Cycle

In the case of the Rankine cycle, the objective function is given in Equation (24). As for the Carnot cycle, both Reynolds numbers are variables of the objective function. In addition, the mass flow rate of the working fluid, here ammonia is also one of the variables. Finally, water temperatures at the outlet of the heat exchangers are needed to calculate the enthalpies required for the objective function as shown in Equations (24)–(26). These temperatures were therefore also set as variables during the optimization process. This led to a total of five variables: Re_ws_, Re_cs_, *T_wso_*, *T_cso_*, and *m_wf_*. As for the Carnot cycle, boundaries were used on the Reynolds number to restrain the water mean velocity between the 0.2 to 1.8 m/s range. Temperatures were taken to be between *T_csi_* and *T_wsi_*. *m_wf_* was restrained to be between 0.001 kg/s and a value that is equal to a water mean velocity of 1.8 m/s, as water mass flow rate is higher than ammonia mass flow rate in OTEC system.

As the optimization is based on enthalpies values, the following constraints were added to ensure that the heat balance (27) and energy conservation (28,29) are respected.

(27) $$C_{ws}\left(T_{wsi}-T_{wso}\right)-C_{cs}\left(T_{cso}-T_{csi}\right)=m_{f}\left(h_{2}-h_{3}-h_{1}+h_{4}\right)$$

(28) $$C_{ws}\left(T_{wsi}-T_{wso}\right)=m_{f}\left(h_{2}-h_{1}\right)$$

(29) $$C_{cs}\left(T_{cso}-T_{csi}\right)=m_{f}\left(h_{3}-h_{4}\right)$$

## 4. Optimization Results

### 4.1. Carnot Cycle Results

**Figure 3:**
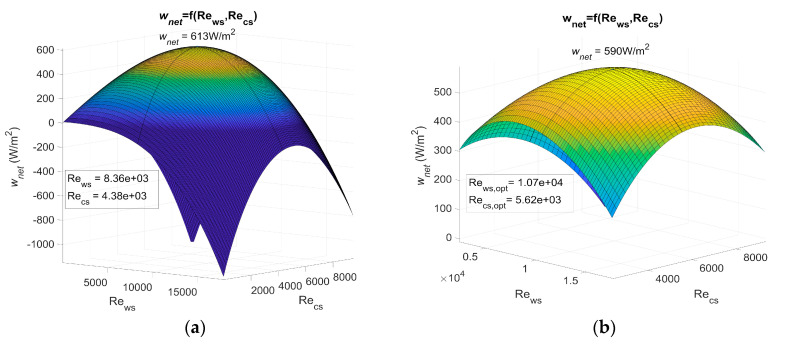

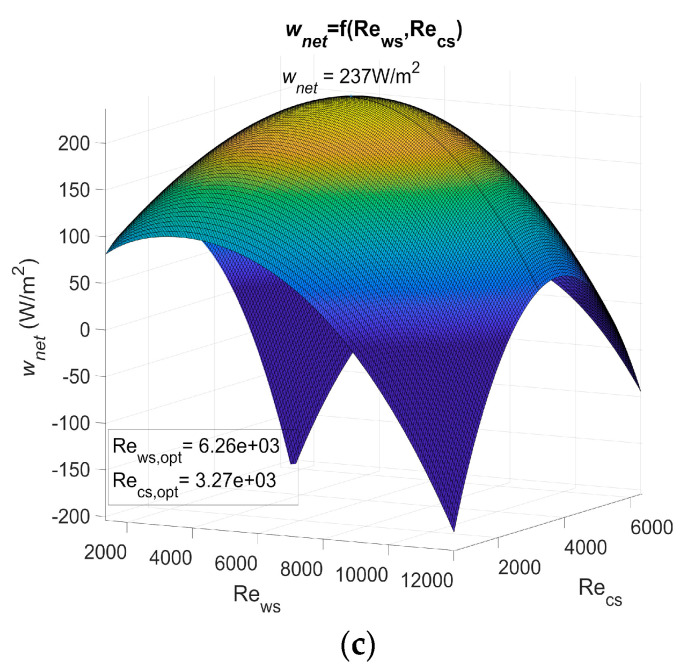
(**a**) Maximum net power output of an OTEC power plant using Plate heat exchanger (PHE) 1 as both evaporator and condenser as a function of Reynolds, (**b**) maximum net power output of an OTEC power plant using PHE 2 as both evaporator and condenser as a function of Reynolds, and (**c**) maximum net power output of an OTEC power plant using PHE 3 as both evaporator and condenser as a function of Reynolds.

Figure 3 shows *w_net_* as a function of the Reynolds number for PHE 1, PHE 2, and PHE 3. Table 3 shows the heat transfer coefficient and pressure drop of the three heat exchangers.

The actual power output of an OTEC power plant using the specified heat exchanger should be less than these results as calculations are based on a Carnot cycle, and if the pressure drop is considered, the required power for water ducting is not. However, the optimum Reynolds numbers *Re_ws,opt_* and *Re_cs,opt_* found in these results show that, for a non-negligible range of Reynolds numbers, the net power output can be null or negative (dark blue areas on Figure 3). The flow rate should then be controlled rigorously to adjust the Reynolds numbers in the heat exchangers.

This work allows the performances of heat exchangers to be compared in terms of the OTEC system’s maximum net power output per unit of heat exchanger surface area, *w_net,max_*. These results show the importance of correctly selecting a heat exchanger, as *w_net,max_* is highly dependent on which one is used. Indeed, a huge difference was noticed between the results using PHE 3 and the results using the other two at their optimal operating point. PHE 1 led to a *w_net,max_* that was 158% higher than the one of PHE 3. As for PHE 2, the *w_net,max_* was 149% higher than the one achieved with PHE 3. This difference can be explained by the low heat transfer coefficient of PHE 3.

PHE 2 led to a *w_net,max_* that was only 3.7% lower than PHE 1, even though its heat transfer coefficient was 35% lower than the one of PHE 1. This can be explained by a friction factor that was 60 to 66% lower in the case of PHE 2. In addition, PHE 2 was found to be less sensitive to a change in the Reynolds numbers. PHE 1, however, presented optimum Reynolds numbers that were 22% lower than those of PHE 2. From these observations, one can easily conceive a heat exchanger that would lead to a higher *w_net,max_* than PHE 1 and also present lower heat transfer coefficient if the pressure drop was low enough. This would be possible because a low pressure drop allows the use of higher Reynolds numbers to compensate for a low heat transfer coefficient. Such a heat exchanger, because of its low pressure drop, would be less affected by a change in the operating Reynolds numbers. A high pressure drop heat exchanger, however, would require relatively lower optimum Reynolds numbers, which implies a lower pumping power for water ducting and/or lower diameter pipes.

**Figure 4:**
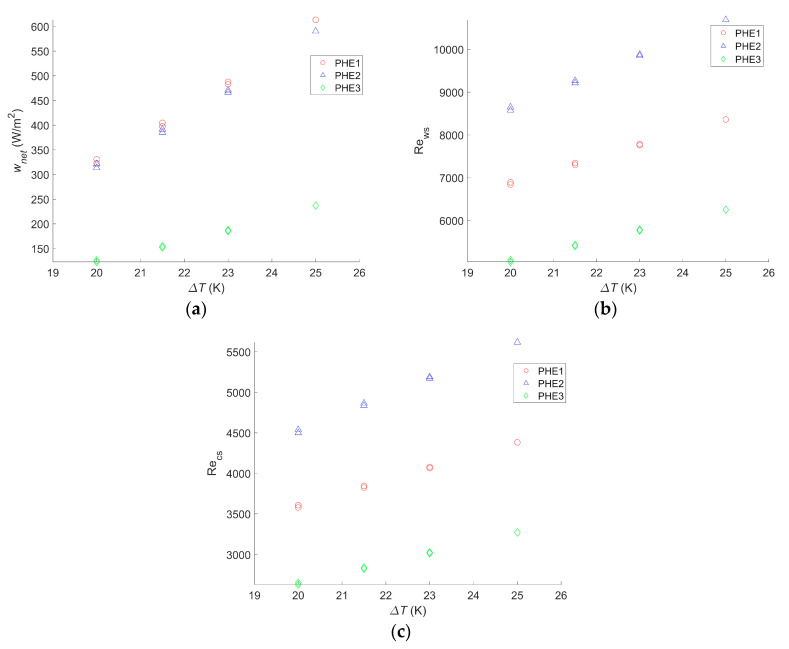
(**a**) Maximum net power output per square meter as a function of the temperature difference between warm and cold water, (**b**) Reynolds number of the warm water inside the evaporator as a function of the temperature difference between warm and cold water, and (**c**) Reynolds number of the cold water inside the condenser as a function of the temperature difference between warm and cold water.

The same calculations were carried out for different hot and cold seawater temperatures to figure out how they would affect the net power output for each heat exchanger. The results are given in Figure 4.

*w_net,max_* will change significantly with a change in the seawater temperature. Indeed a 20% drop in *w_net,max_* was observed for the three heat exchangers when a 2 °C change in the seawater source occurred. Yeh et al. found a decrease of 35% for the same temperature drop in the cold seawater, but warm seawater was fixed at 25 °C, instead of 30 °C in the present study [30]. Sinama et al. found a 40% decrease when the warm seawater temperature dropped from 28 °C to 25 °C against 32% in this study for the same seawater temperature values [17]. Additionally, for the same temperature change, Uehara and Ikegami showed a decrease of 44% in the net power output of the OTEC power plant [23]. In their paper, VanZwieten et al. showed a decrease of 20% and 16% when the temperature changed from 20.12 °C to 21.72 °C and from 20.12 °C to 21.37 °C, respectively, against a 17% drop in this study when a 1.5 °C change occurred [31]. For a change of 5 °C in the seawater source temperature, the calculated drop in *w_net,max_* was 45%. The difference in the decrease of the net power output with other studies can be explained by the fact that the authors considered water ducting as well as the working fluid circulation pump [17,23,30,31]. Therefore, when the temperature difference decreases, the gross power output greatly decreases, whereas losses due to pumping and pressure drop hardly change. The impact seems to be the same if the change consists of a decrease in the warm seawater source temperature or an increase of the cold seawater source temperature.

Although the Reynolds numbers for PHE 1 were still 20% to 22% lower than those of PHE 2, the optimal operating point also varied with the temperature change; the bigger the water temperature difference is, the more an increase of the flow rate will result in an increase of the heat exchange. However, a seawater temperature change only has a limited impact on the friction factor.

Furthermore, as the maximal power output decreases with the temperature change, the range of operating points resulting in a positive net power output decreases as well.

Finally, if a change of the seawater temperature does not affect which heat exchanger leads to the highest *w_net,max_*, the change in the operating points and the increase of the negative power output operating conditions make the flow rate monitoring even more important if the system is to be installed where the water condition changes throughout the year. In addition, for an installation where the seawater temperature does not vary, calculations need to be adjusted to the actual condition to find the optimal operating point.

### 4.2. Rankine Cycle Results

The results for PHE 1, PHE 2, and PHE 3, studied by Kushibe et al. [28], gave *w_net,max_* values that were, as expected, lower than those using the Carnot cycle, as shown in Table 4.

Moreover, the results showed a difference between the optimal operating point for the same heat exchanger, especially for the Reynolds number of warm seawater. This suggests that calculations should be done for the actual cycle used in each plant to find the optimized operating point.

**Figure 5:**
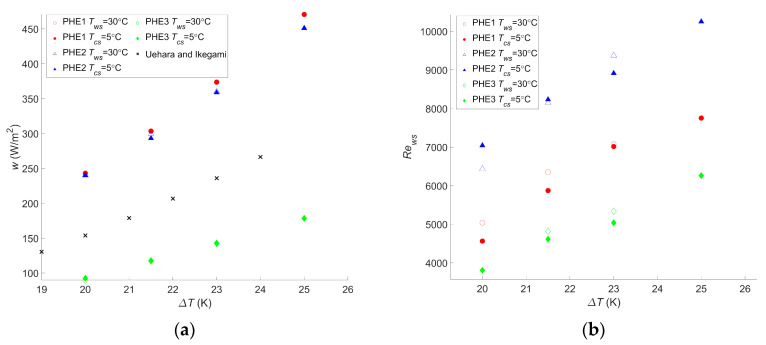

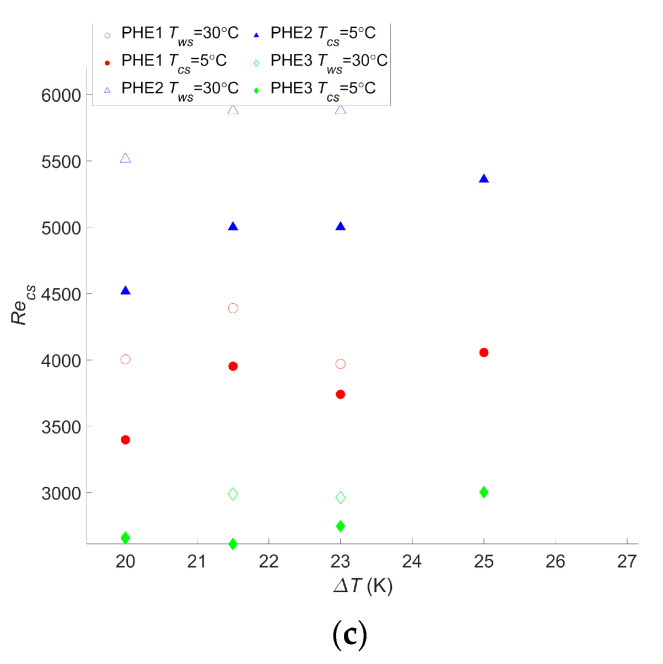
(**a**) Maximum net power output per square meter as a function of the temperature difference between warm and cold water, (**b**) Reynolds number of the warm water inside the evaporator as a function of the temperature difference between warm and cold water, and (**c**) Reynolds number of the cold water inside the condenser as a function of the temperature difference between warm and cold water.

For this cycle as well, the calculations were realized for different temperatures, and the results are given in Figure 5.

These results presented a *w_net,max_* ranging from 90 W/m^2^ to 471 W/m^2^ for PHE 3 at the lowest temperature difference and PHE 1 at the highest temperature difference, respectively. As a comparison, the study performed by Uehara et al. presented a net power output of 153.9 W/m^2^ at a temperature difference, Δ*T*, of 20 °C and a net power output of 236 W/m^2^ at a Δ*T* of 23 °C [23]. For the same Δ*T*, the performances of the two heat exchangers they considered were between those of PHE 3 and PHE 2, giving a *w_net,max_* of 90 W/m^2^ and 240 W/m^2^, respectively, at a Δ*T* of 20 °C, and 143 W/m^2^ and 360 W/m^2^, respectively, at a Δ*T* of 23 °C. These values are rather low but are still in the range of what was found in the current study. Moreover, it should be noted that, in their paper, they considered the pumping power required for water ducting, working fluid pumps, and the heat transfer coefficient of the working fluid, which explains the lower power output.

Bernardoni et al. showed a net power output of 167 W/m^2^ and a gross power output of 278 W/m^2^ for a Δ*T* of 24 °C in their optimization using plate heat exchangers [16]. This was lower than the 293 W/m^2^ found with PHE 2 at a Δ*T* of 21.5 °C. In their work, the authors considered water ducting, ammonia pumping, and the ammonia heat transfer coefficient, although only the latter contributed to decreasing the gross power output.

Both these studies are more accurate than the presented one; their goal was to precisely assess the net power output of the system, which was either to calculate a reliable LCOE in the case of Bernardoni et al. or for a very specific OTEC power plant design in the case of Uehara et al. The current study, however, focused on comparing the performances of heat exchangers in terms of the OTEC system performance, and such accurate assessments are less relevant in this case. In comparison, in the other two studies, there is still plenty of room for increasing the OTEC system performance regarding the choice or optimization of the heat exchanger. Regardless, the trend obtained by Uehara and Ikegami is very consistent with what was found in this work, as can be seen in Figure 5a.

As for the Carnot cycle, the results for the Rankine cycle indicate that a temperature change will have a significant impact on the maximum net power output of the OTEC power plant. A 20% decrease in *w_net,max_* occurred with a decrease of two degrees in the temperature difference, and a 47%–50% decrease was observed with a decrease of five degrees in the temperature difference, depending on the heat exchanger. These changes were the same as those observed with the Carnot cycle. For this cycle too, a change in the water temperature will not affect which heat exchanger is the most suitable even if the gap between each heat exchanger performance can vary along with the temperature difference. Moreover, although it is not as clear as for the Carnot cycle calculation, the Reynolds numbers of the optimized operating points tend to increase with the temperature difference. This confirms that calculations are needed for each application and its specific operating condition to find the optimal operating point. It also confirms that the flow rate should be monitored and adapted in case a change of temperature occurs. The difference between the Reynolds numbers for PHE 1 and PHE 2 remains close to the results for the Carnot cycle, with values 21% to 35% lower for PHE 1.

Although the maximum net power output and operating points vary from the Carnot cycle to another, the preferable heat exchanger remains the same. Indeed, the ratio of *w_net,max_* achieved using a Rankine cycle over the one achieved using the Carnot cycle was found to be fairly constant, with figures ranging between 0.73 and 0.78 for all heat exchangers and for all the computed Δ*T*. It is therefore possible, for the selection of the heat exchangers, to base the calculation on the Carnot cycle only.

In the case of an OTEC power plant, pressure drop plays a major role in the choice of a heat exchanger. Indeed, a study that only considers heat transfer performance might reach a different conclusion regarding which heat exchanger is the most suitable one. In such a study, the net power output would be found to increase with the Reynolds number without any limitation. Therefore, the optimum Reynolds numbers would be the maximum ones reachable for the corresponding heat exchanger and would not vary with a change in water temperature as they do with the presented results in Figure 4 and Figure 5. The limitation induced by the pressure drop that occurs within the heat exchangers can easily be seen in the Carnot cycle results: without pressure drop consideration, the net power output cannot be negative nor decrease as the Reynolds number increases. However, Figure 3 presents parabolic graphs that include areas of negative net power output.

## 5. Conclusions

This work aimed to compare the performance of heat exchangers according to the OTEC system’s maximum net power output. For both the Carnot and Rankine cycles, an objective function of the net power output per unit of heat exchanger surface area was defined. The objective functions took into account the pressure drop that occurred in the heat exchangers. These functions were maximized using the derivative-free method implemented in Matlab. The calculations were realized for three different heat exchangers at four temperature differences; each value of temperature difference included two points and considered an increase of the deep seawater temperature or a decrease of the warm seawater temperature. The evaluation method that was developed differs from others due to its relative ease of use. The following conclusions were made.

- For the sole purpose of a heat exchanger comparison, calculations based on the Carnot cycle for any source temperature were sufficient, as the cycle and temperature difference do not have an impact on the choice of the heat exchanger even though they do change the power output and optimized operating conditions. The Rankine cycle calculations presented a maximum net power output 23–27% lower than for the Carnot cycle that dropped ~10% each time a temperature difference decrease of 1 °C was observed. The evolution of the power output as a function of the temperature difference was found to follow the same trends as found in other studies.
- The maximum net power output was found to highly depend on the chosen heat exchangers. For the highest temperature difference, the most suitable heat exchangers among the three considered led to a maximum power output 158% and 165% higher than the worst heat exchanger for the Carnot and Rankine cycles, respectively.
- Due to the trade-off that exists between the heat transfer coefficient and the pressure drop, the heat exchanger presenting the highest heat transfer coefficient is not necessarily the one that will lead to the highest maximum net power output. In this study, for the Carnot cycle, PHE 2 competed with PHE 1 as it led to a maximum net power output that was only 3.7% lower than the one of PHE 1, even though its heat transfer coefficient was 35% lower.
- Heat exchangers with a high pressure drop and those with a low pressure drop have been found to have their own advantages and drawbacks. High pressure drop heat exchangers require lower Reynolds numbers, and therefore a smaller pumping power and/or a smaller pipe diameter are needed. Low pressure drop heat exchangers are less sensitive to a change in the Reynolds numbers, which can be useful in case a change in the operating conditions is needed. This is even more important as the results showed that negative net power output can be reached for low enough Reynolds numbers.

If the most suitable heat exchanger was found, assessments of the maximum power output and the optimum operating point are not accurate enough to be used for OTEC design. Working fluid heat transfer coefficient as well as fouling thermal resistance were neglected; however, as they can have a significant impact on the OTEC performance, they should be further studied in the future. In addition, the comparison can only be done with existing heat exchangers, as specific correlations for the heat transfer coefficient and the pressure drop are needed; thus, it is difficult to know in advance what the effect of a specific design will be. The next step is to improve the accuracy of this method by including the heat transfer coefficient of the working fluid, the pumping power for water ducting, and more specific cycles.

## Figures and Tables

**Table 1:** Heat exchanger specifications. PHE: Plate heat exchanger.

Heat Exchanger	PHE 1	PHE 2	PHE 3
Length *L* (mm)	960	718	1765
Width *Wi* (mm)	576	325	605
Thickness *t* (mm)	0.7	0.5	0.6
Space between plates *δ* (mm)	4.00	3.95	2.68
Equivalent diameter *D* (mm)	8.00	7.90	5.36
Material	SUS316	Titanium	Titanium
Thermal conductivity λ_p_ (W/(m·K))	16.3	21	21
Pattern	Herringbone (72°)	Herringbone (30°)	Fluting and drainage
Number of plates	120	20	52
Total heat transfer area *A* (m^2^)	100.3	3.96	40.6
Total cross surface area *S* (m^2^)	0.140	0.012	0.041

**Table 2:** Coefficients for the Nusselt numbers and friction factor correlations.

Heat Exchanger	*d*	*γ*	*n*	*β*	*ξ*
PHE 1	0.111	0.8	1/3	1.4863	−0.0540
PHE 2	0.058	0.8	1/3	6.5059	−0.3292
PHE 3	0.051	0.8	1/3	0.7371	−0.1274

**Table 3:** Heat transfer coefficient and friction factor of the seawater inside the heat exchangers.

Heat Exchanger	*α_ws_* (W/m^2^·K)	*α_cs_* (W/m^2^·K)	*f_ws_* (−)	*f_cs_* (−)
PHE 1	20 569	14 450	0.913	0.945
PHE 2	13 259	9 334	0.307	0.379
PHE 3	11 191	7 843	0.242	0.263

**Table 4:** Comparison between the Carnot and Rankine cycles.

	PHE 1		PHE 2		PHE 3	
	Carnot	Rankine	Carnot	Rankine	Carnot	Rankine
*w_net_* (W/m^2^)	613	471	590	451	237	178
Re_ws_	8361	7876	10,690	10,121	6255	5753
*V_ws_* (m/s)	0.837	0.788	1.08	1.03	0.934	0.859
Re_cs_	4383	4381	5618	5762	3274	3277
*V_cs_* (m/s)	0.832	0.831	1.08	1.11	0.927	0.928

## Nomenclature

Nomenclature		
*A*	[m^2^]	Heat transfer surface area
*As*	[m^2^]	Heat exchanger surface area
*B*	[K/W]	$\frac{plate\;thickness}{\lambda_{plate}A}$
*Cp*	[J/(kg·K)]	Specific heat
*C*	[W/K]	$\dot{m}Cp$ heat capacity
*D*	[m]	Equivalent diameter
*f*	[-]	Friction factor
*h*	[J/kg]	Specific enthalpy
*L*	[m]	Length of the plate
*m*	[kg/s]	Mass flow rate
Nu	[-]	Nusselt number
*P*	[kPa]	Pressure
Pr	[-]	Prandtl number
*Q*	[W]	Heat transfer rate
Re	[-]	Reynolds number
*R_f_*	[m^2^·K/W]	Resistance due to fouling
*S*	[m^2^]	Total cross-sectional surface area
*s*	[J/(kg·K)]	Specific entropy
*T*	[K]	Temperature
*U*	[W/(m^2^·K)]	Overall heat transfer coefficient
*v*	[m/s]	Mean velocity
*Wi*	[m]	Width of the plate
*W*	[W]	Power output
*w*	[W/m^2^]	Power output per unit of surface area
*x*	[-]	Vapor quality
*α*	[W/(m^2^·K)]	Heat transfer coefficient
Δ*T*	[K]	Temperature difference
*λ*	[W/(m·K)]	Thermal conductivity
*Λ*	[-]	Lagrange multiplier
*μ*	[Pa·s]	Dynamic viscosity
*ρ*	[kg/m^3^]	Density
*τ*	[Pa]	heat exchanger wall shear stress

Subscripts	
*cs*	Cold source
*csi*	Cold source inlet
*cso*	Cold source outlet
*gross*	Gross
*in*	Inlet
*max*	Maximum
*net*	Net
*opt*	Optimum
*out*	Outlet
*p*	Plate
*w*	Water
*ws*	Warm source
*wsi*	Warm source inlet
*wso*	Warm source outlet
*wf*	Working fluid

## Appendix A

This appendix shows details on how the following equation can be derived,

(A1) $$T_{2}=\frac{T_{wsi}-T_{wso}e^{{\mathrm{NTU}}_{\mathrm{ws}}}}{\epsilon_{ws}}\;and\;T_{4}=\frac{T_{csi}-T_{cso}e^{{\mathrm{NTU}}_{\mathrm{cs}}}}{\epsilon_{cs}}$$

where *T*_2_ and *T*_4_ are the temperatures on the corresponding points in Figure 2.

The heat exchanged in the condenser, *Qcs*, is equal to:

(A2) $$Q_{cs}=mc_{p_{cs}}\left(T_{cso}-T_{csi}\right)$$

Using the logarithmic mean temperature difference method, the same heat can be expressed as the following:

(A3) $$Q_{cs}=UA\Delta T_{LMTD}$$

where:

(A4) $$\Delta T_{LMTD}=\frac{(T_{3}-T_{cso}-\left(T_{csi}-T_{4}\right)}{\ln\left(\frac{T_{3}-T_{cso}}{T_{4}-T_{csi}}\right)}$$

Given that *T*_3_ = *T*_4_, assuming the fluid is at a saturated state at the inlet and outlet of the condenser, Equation (A3) becomes:

(A5) $$Q_{cs}=UA\frac{T_{csi}-T_{cso}}{\ln\left(\frac{T_{4}-T_{cso}}{T_{4}-T_{csi}}\right)}$$

From Equations (A2) and (A5), the equation is:

(A6) $$UA\frac{T_{csi}-T_{cso}}{\ln\left(\frac{T_{4}-T_{cso}}{T_{4}-T_{csi}}\right)}=-mc_{p}{}_{cs}\left(T_{csi}-T_{cso}\right)$$

and therefore:

(A7) $$\exp\left(\frac{UA}{mc_{p}{}_{cs}}\right)=\frac{T_{4}-T_{cso}}{T_{4}-T_{csi}}$$

NTU_cs_ and ε_cs_ are defined by [25]:

(A8) $${\mathrm{NTU}}_{\mathrm{cs}}=\frac{UA}{{\mathrm{mC}}_{p}{}_{cs}}\;and\;\epsilon_{cs}=1-e^{-NTU_{cs}}$$

*T*_4_ can be deduced from Equation (A7) as:

(A9) $$T_{4}=\frac{T_{csi}-T_{cso}e^{{\mathrm{NTU}}_{\mathrm{cs}}}}{\epsilon_{cs}}$$

For the evaporator, the assumption is that *T*_1_ = *T*_2_ is made, and then the calculations are the same. Equation (A10) is used:

(A10) $$Q_{ws}=UA\frac{(T_{wsi}-T_{2}-\left(T_{wso}-T_{1}\right)}{\ln\left(\frac{T_{wsi}-T_{2}}{T_{wso}-T_{1}}\right)}=UA\frac{\left(T_{wsi}-T_{wso}\right)}{\ln\left(\frac{T_{wsi}-T_{2}}{T_{wso}-T_{2}}\right)}=mc_{p}{}_{ws}\left(T_{wsi}-T_{wso}\right)$$

NTU_ws_ and ε_ws_ are defined the same way:

(A11) $${\mathrm{NTU}}_{\mathrm{ws}}=\frac{UA}{{\mathrm{mC}}_{p}{}_{ws}}\;and\;\epsilon_{ws}=1-e^{-NTU_{ws}}$$

Thus, *T*_2_ can be expressed as:

(A12) $$T_{2}=\frac{T_{wsi}-T_{wso}e^{{\mathrm{NTU}}_{\mathrm{ws}}}}{\epsilon_{ws}}$$
